# Entropy and Polarity Control the Partition and Transportation of Drug-like Molecules in Biological Membrane

**DOI:** 10.1038/s41598-017-18012-7

**Published:** 2017-12-18

**Authors:** Qiang Zhu, Yilin Lu, Xibing He, Tao Liu, Hongwei Chen, Fang Wang, Dong Zheng, Hao Dong, Jing Ma

**Affiliations:** 1 Kuang Yaming Honors School, Nanjing University, Nanjing, 210023 P. R. China; 2 Institute of Theoretical and Computational Chemistry, School of Chemistry and Chemical Engineering, Nanjing University, Nanjing, 210023 P. R. China; 3 School of Pharmacy, University of Pittsburgh, 3501 Terrace Street, Pittsburgh, PA 15213 USA; 4 College of electronic information engineering, Sanjiang University, Nanjing, 210012 P. R. China

## Abstract

Partition and transportation of drug in the plasma membrane of a mammalian cell are the prerequisite for its function on target protein. Therefore, comprehensive understanding of the physicochemical properties and mechanism behind these complex phenomena is crucial in pharmaceutical research. By using the state-of-art molecular simulations with polarization effect implicitly or explicitly included, we studied the permeation behavior of 2-aminoethoxydiphenyl borate (2-APB), a broad-spectrum modulator for a number of membrane proteins. We showed that the protonation state and therefore the polarity of the drug is critical for its partition, and that the drug is likely to switch between different protonation states along its permeation pathway. By changing the degrees of freedom, protonation further affects the thermodynamic of the permeation pathway of 2-APB, leading to different entropic contributions. A survey on 54 analog structures with similar backbone to 2-APB showed that delicate balance between entropy and polarity plays an important role in drugs’ potency.

## Introduction

The capacity of an ideal drug to move across membranes is an extremely important factor affecting its absorption, distribution, potency, and elimination^1^. For small molecule drugs, passive diffusion is the major way to penetrate through the lipid membrane^2^. The property of membrane permeability was found to be a key factor in the drug design process of small molecules^3,4^. Therefore, the ability to predict drugs’ membrane permeability prior to synthesis with high accuracy is crucial in the early stages of drug design. Comprehensive knowledge of the physicochemical properties and mechanism underlying these complex phenomena will be of great help. For example, the famous “Rule of 5” proposed by Lipinski *etc*. identified the most relevant physicochemical parameters that determines membrane permeability, including lipophilicity, polarity and size of drug-like compounds^5^. However, how these interrelated factors synergistically affecting the performance of drugs remains unclear.

**Figure 1:**
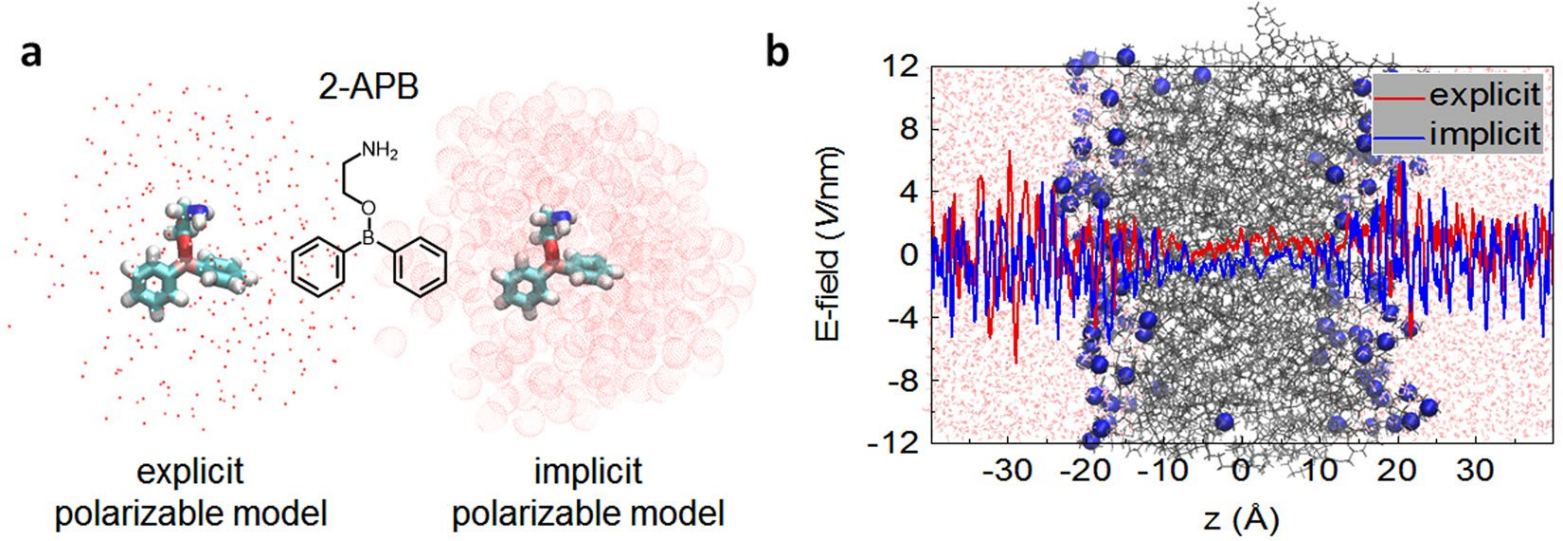
The polarizable models used in this work. (**a**) The explicit and implicit polarizable models, and a stick representation of the 2-APB molecule in the neutral form. In both models, the partial charges of 2-APB were determined in response to the membrane environment, but with different parametrization procedures. (**b**) The calculated dielectric profiles in the fully hydrated POPC lipid bilayers with both models are qualitatively consistent with each other. The x-axis, z, denotes the position with respect to the center of the lipid bilayers.

2-aminoethoxydiphenyl borate (2-APB, Fig. 1a) is a membrane-permeable, broad-spectrum modulator for a variety of membrane proteins^6–8^. Especially for the calcium signaling through store-operated calcium (SOC) channels^9^, great efforts have been made to reveal that the function of 2-APB is concentration dependent: potentiation of the SOC entry at low concentration (1–20 *μM*), but inhibition at high concentration(25–100 *μM*)^9–11^. Electrophysiological and biochemical measurements suggested that the inhibition by 2-APB happens at the SOC channel, the channel’s activator protein, as well as the coupling between the two^12^, while potentiation on SOC entry is because of the 2-APB induced pore expansion^13^. All of these features make SOC a unique target for 2-APB. Given the pieces of information reported, however, the whole picture about the regulation mechanism of 2-APB on the SOC entry at atomic level still remains elusive^9^. Experimentally, the p*K*_a_ of 2-APB was determined to be 9.6 in water^14^, suggesting that most of 2-APB molecules in water are protonated. But it was found to be deprotonated in aprotic solvent^15^.

Based on the first principle calculations, we found that the favorable intra- and inter-molecular interactions could be formed between the boron center and the nitrogen atom on the amine group in 2-APB, and therefore we speculated that the dual functionality of 2-APB is likely to be attributed to the different binding sites with different functional forms of the drug^16^. As SOC channels being located in the plasma membrane, however, the environment was not explicitly considered in the previous work. Seemingly, knowledge about the interaction between 2-APB and membrane is the prerequisite for the understanding of its regulation mechanism on calcium entry pathway.

Extensive studies have been done to explore the transportation of drug-like compounds with computations, which provide valuable insights into the details at atomic level that cannot be accessed by experiments^17–20^. Among different descriptors for characterizing the permeability of drug-like compounds^21–23^, the partition coefficient (log*P*) is the most commonly used one. Log*P* is defined as the logarithm of the ratio of compounds dissolved in lipid bilayers and the buffer water of a two-compartment system under equilibrium conditions. However, due to the difficulties in laboratory measurements, in practice the N-octanol/water partition coefficient was commonly used as a surrogate, since the N-octanol and membrane lipids share similar amphiphilic feature^24^. Different structure- or property-based methods have been developed to fast predict log*P* of large amount of molecules^25^. On the other hand, log*P* could be calculated from the transfer free energy between polar and non-polar environments in a diphase system by using molecular dynamics (MD) simulations^26^. It should be mentioned that free energy based evaluation of permeability is computationally more expensive but has been found to be clearly superior to those empirical protocols, as substantial improvement on accuracy was observed^3^.

Another important factor for *in silico* prediction of drug permeation is how to deal with the polarization effect, especially at the interfacial region between water and lipid bilayers. Due to the inhomogeneous nature of cell membrane, its interior and exterior have quite different dielectric constant conditions^27^. Theoretical model based on fitting the fluctuation of local field to the polarization response calculated with the Poisson’s equation shows that, the permittivity profile of membrane system changes as a function of position^28^. Therefore the membrane environment has distinct modulations on molecules. For example, the shift of p*K*_a_ value for titratable groups in response to the environment is not unusual^29^, and the binding affinity between drug and membrane protein was found to be lipid-component dependent^30^. Great efforts have been made to develop polarizable models^31^, such as Drude^32,33^, AMOEBA^34–36^, X-POL^37^, etc, especially those relevant to lipid bilayers^38,39^.

In the present work, we used computer simulations to study the behavior of 2-APB in fully hydrated 1-palmitoyl-2-oleoyl-sn-glycero-3-phosphocholine (POPC) lipid bilayers. The observations from MD simulations with polarization effect taken into account, interpreted with the help of thermodynamics and kinetics analysis, have defined the contributions of key factors, in particular the entropy and polarity for regulating the membrane permeation of drug-like compounds. We further explored 54 compounds based on the mother structure 2-APB, and found delicate balance between the two factors, therefore may provide insights into drug design with higher efficiency.

## Computational Details

Here we present a brief description of the protocol used in this work. More details about the polarizable models, the parameterization procedure, as well as other computational details could be found in the Supporting Information (Supplementary Figures S1–S7). To take the polarization effect from the membrane environment into account, based on the conventional CHARMM force field, both the implicit and explicit polarizable models were used to update the partial charge of the 2-APB molecules (Fig. 1a).

The general idea in the explicit polarizable model is that, the partial charges of a molecule were not fixed but were changeable in response to the local condition represented with background point charges^40–42^. In contrast, the partial charge in the implicit polarizable model was fixed, but the parameterization was carried out to take the surrounding environment into consideration by using the continuum medium model, and ensemble average of the trajectory (obtained from MD simulations by using conventional force field) was used to account for the redistribution of molecular dipole (Supplementary Schemes 1 and 2). Similar protocol was adopted to study the partition of small molecules in biological membrane^43,44^.

Unbiased molecular dynamics simulations (with the total time length of 1.56 μs) and free energy calculations (a total of 28.8 μs) were then used to explore the partition and transportation of 2-APB with different protonation states in POPC. Based on the free energy profiles for both the neutral and charged forms, a thermodynamic cycle was employed to estimate the p*K*_a_ shift of 2-APB at different insertion depths in the membrane. The free energy profiles were further decomposed into contributions from entropy and internal energy. Finally, we studied 54 drug-like compounds based on 2-APB’s backbone structure, and found the delicate balance between key factors for their functions.

## Results and Discussion

### Implicit and explicit polarizable models have essentially the same description for the dielectric profile in biological membrane

Due to the profound polarization effects at the interfacial region of the membrane, the performance of the implicit and explicit polarizable models for describing the inhomogeneous system was tested.

The calculated local electric field taken from the ensemble average of the equilibrated trajectory from both models is shown in Fig. 1b. As pointed by Zhou etc^28^. and our previous work^45^, the dielectric constant of the system is related to the local induced field and the position. In this work, the amplitude of the oscillation of the local electric field in bulk aqueous phase is 6–8 times larger than that in the membrane center while the oscillation drops to much lower level in the hydrophobic core, showing the heterogeneity of membrane: the exterior has a high dielectric constant environment with strong polarizability and the interior has much less polarity. The net oscillation, however, is close to zero by cancelling the positive and negative directions of oscillation. More importantly, the profiles in both models are almost indistinguishable.

It seems that both implicit and explicit polarization protocols could well capture the characteristics of the local dielectric constant of the system, while the former is expected to be more computationally economical. We then further tested the performance of two models to predict the partition of drug in the membrane, as shown below.

### MD simulations reproduce the experimental log*P* of 2-APB

The partition behavior of both the neutral and positively charged forms were studied by performing unrestrained MD simulations. To account for the polarization of the membrane environment, both the implicit and explicit polarizable models were used.

**Figure 2:**
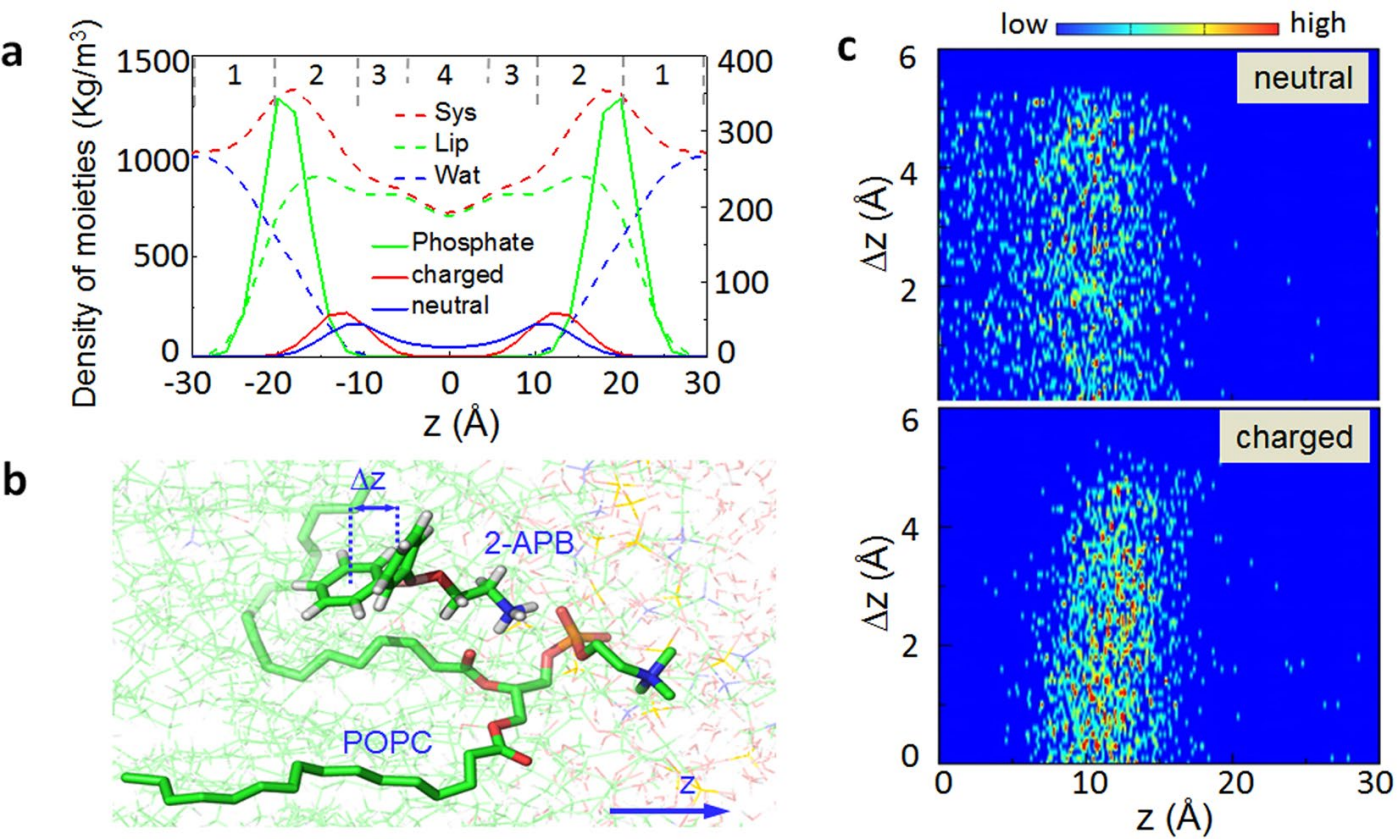
The partition of 2-APB in a POPC lipid bilayers. (**a**) Density distribution profiles of different species. The four regions are shown by the numbers on the top. The scale of y-axis on the left is for the density of the system, the lipid bilayers and waters, while on the right is for phosphate atom and 2-APB. (**b**) A representative snapshot showing the specific interaction between the amine group of 2-APB and the phosphate group on POPC lipid. Δz is the difference between the coordinate of two benzene rings of 2-APB along the z-axis shown in the lower-right panel. (**c**) The unsynchronized insertion of two phenyl groups in the neutral (upper panel) and charged (lower panel) forms of 2-APB, as shown by the distributions of Δz at different positions along the membrane normal z. Data in both panels were normalized for comparison.

In the view of the inhomogeneity of the system we construct, a so-called “four region model”^46–48^ is employed (Fig. 2a). Region 1: starts from the bulk water and end at where the density of water and lipid are comparable; region 2: the region of the head-groups of the POPC until the density of water is <1%; region 3: high-density tail region; region 4: low-density tail region.

Starting from an initial configuration where half of the drugs were in the bulk water and the rest were in the membrane (Supplementary Figure S1), the drugs prefer to stay in the membrane environment within sub-microsecond simulations, and the neutral 2-APB is more close to the center of the membrane, while the charged ones were ejected from the membrane (Fig. 2a). The amine group on the polar head of the drug has specific interaction with the phosphate group of the lipid (Fig. 2b), while the two phenyl rings insert into the hydrophobic fatty acid chains of the membrane. Interestingly, one of the two rings firstly inserts into the hydrophobic core of lipid, anchoring the drug molecules nearby the membrane-water interface (Fig. 2c). The second ring could then easily enter the hydrophobic region.

The partition coefficients derived from the simulation are present in Table 1. Based on the observations from the present sub-microsecond unbiased simulations, the calculated log*P* value (the definition is shown in Supplementary Information) converged very slowly. After ~800 ns MD simulations for both the implicit and explicit polarizable models, they gave a nearly equilibrated log*P* close to the range of 2.1–2.6 determined by experiments^8^. Presumably, extending the MD simulations to longer timescale could introduce a subtle change of the log*P*. To be specific, the data for both polarizable models fall into the range of experimental data by extrapolating the simulation time to several μs. However, fully converged results may need even much longer time mainly because of the delicate balance of drug distribution in two phases.

**Table 1:** The calculated log*P* of 2-APB derived from MD simulations by using two polarizable models.

Simulation time (ns)	log*P*		
	Explicit	Implicit	Exp.
300	1.851 ± 0.009	1.530 ± 0.017	2.1–2.6
350	1.906 ± 0.014	1.584 ± 0.014	
400	1.946 ± 0.013	1.636 ± 0.012	
700	1.891 ± 0.006	1.815 ± 0.005	
740	1.911 ± 0.006	1.826 ± 0.005	
780	1.929 ± 0.007	1.842 ± 0.004	

In conclusion, both models are capable of predicting the partition coefficient of drug, though the explicit model is much more computational demanding than the implicit one. For the purpose of high-throughput and efficient simulations, in the following section, the implicit polarizable model was used to calculate the free energy profile for drug permeation.

### The neutral 2-APB is energetically more favorable than its charged form to insert into membrane

**Figure 3:**
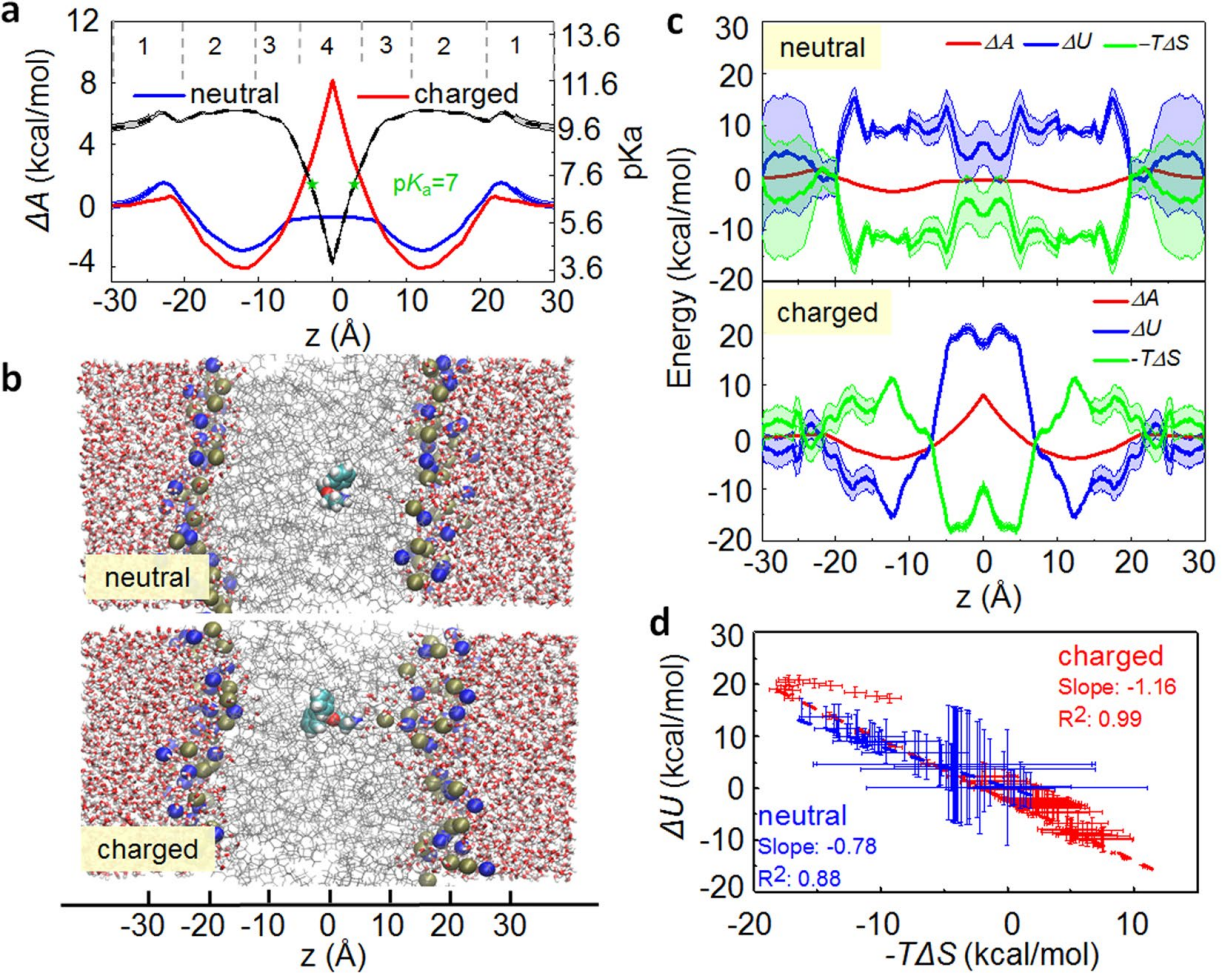
The permeation free energy of 2-APB in a fully hydrated POPC lipid bilayers. (**a**) The free energy profiles of the neutral (blue line) and charged (red line) forms of 2-APB in a POPC bilayer, and the predicted p*K*_a_ of 2-APB at different position of the system (black line). (**b**) Representative snapshots for the two protonated states during permeation, and membrane deformation was observed in the system containing charged 2-APB. (**c**) Decomposition of the Helmholtz free energy (∆*A*, red line) into contributions from internal energy (∆*U*, blue line) and entropy (−*T*∆*S*, green line). (**d**) Correlation between ∆*U* and −*T*∆*S*. The shadow in (**a**), (**c**) and bars in (**d**) represent the standard error.

To quantitatively understand the energy cost of the drug across the membrane environment, advanced sampling technique with implicit polarizable model was used. By using the bulk water as a reference, we found that the neutral form of 2-APB is lipophilic, which has a more favorable permeation pathway than its charged counterpart (Fig. 3a).

The neutral form has a major barrier of ~2 kcal/mol at the position just above the phosphate group, which is consistent with the experimental observations that 2-APB is membrane-penetrable^49^. A plateau could be observed at the center of membrane where the free energy is ~1 kcal/mol lower than that in bulk water, showing that it is energetically favorable for the neutral drug to stay at the interior of the membrane. The charged one, on the other hand, shows a much higher barrier (8 kcal/mol) at the center of the lipid bilayer with respect to the bulk water. Furthermore, due to the favorable electrostatic interactions between the amine group and the phosphate group on lipids, a ~12 kcal/mol barrier could be observed to prevent the translation from one leaflet to the other side. Therefore, protonation state of 2-APB plays a critical role in regulating its permeation through the membrane.

Representative snapshots for both forms of 2-APB in membrane (Fig. 3b) clearly explain the difference: the insertion of the charged drug into hydrophobic core is associated with dehydration of the amine group, and leads to hydrophobic mismatch between the charged group and the alkyl tails. Consequently, deformation of the lipid bilayer around the charged moiety could partially compensate for the energy cost imposed by the membrane, and several water molecules penetrate into the hydrophobic region of the membrane to protect the positively charged amine group. This membrane deformation induced by the insertion of charged group has been observed by others as well^18,46,50–52^. In contrast, the presence of the neutral form inside the membrane does not significantly perturb the local structure of membrane.

### The shift of p*K*_a_ value of 2-APB along with its insertion

Given the different free energy profiles for permeation, it is very likely that 2-APB experiences deprotonation during its penetration into the membrane. The p*K*_a_ of 9.6 indicates that most of 2-APB molecules in water are protonated^14^. The inhomogeneous nature of membrane, however, is likely to modulate the protonation state of the titratable amine nitrogen. So the thermodynamic cycle was employed to estimate the p*K*_a_ shift of 2-APB at different membrane insertion depths (Figure S5). Similar protocol was used to predict the p*K*_a_ shifts of titratable residues^51,53,54^.

The p*K*_a_ of 2-APB varies non-monotonically along with its position (Fig. 3a): slightly upward shift of p*K*_a_ value could be observed at the membrane-water interface, mainly because of the favorable binding between amine and the negatively charged phosphate group. However, a sharp decrease of p*K*_a_ to ~4.0 was found upon membrane internalization. This could be attributed to the incompatibility between the charged amine group and the hydrophobic core of membrane. The position at z = ~4 Å, within the low-density tail region, is a critical point where the predicted p*K*_a_ of 2-APB is 7.

In short, the hydrophobic membrane core region favors the neutral form of 2-APB but disfavors the charged one, and it is reasonable to conjecture that the neutral form is dominant in the membrane, though at the membrane-water interface, the charged form is still the major species.

### Entropy is a key factor for 2-APB’s partition

In order to better understand the process of partition in the membrane, the permeation free energy of 2-APB was carried out at another two different temperatures (305 K, 315 K). All of the three free energy profiles are shown in Figure S6. As the NVT ensemble is used for simulations, the obtained Helmholtz free energy (*A*) could be decomposed into entropic (*S*) and internal energy (*U*) components based on the following equations^55^, where *T* is the temperature, *p* is the pressure, *V* is volume of the system, *μ* is the chemical potential, and *N* is the particle numbers.

1 $$dA=-S\cdot dT-p\cdot dV+\mu \cdot dN$$

2 $$\begin{array}{rcl}-T\cdot S & = & -T\cdot (-\frac{dA}{dT})=T\cdot (\frac{dA}{dT})\approx \frac{T}{2\cdot {\rm{\Delta }}T}({A}_{T+{\rm{\Delta }}T}-{A}_{T-{\rm{\Delta }}T})\\ -T\cdot {\rm{\Delta }}S & = & \frac{T}{2\cdot {\rm{\Delta }}T}({\rm{\Delta }}{A}_{T+\Delta T}-{\rm{\Delta }}{A}_{T-{\rm{\Delta }}T})\end{array}$$

The neutral and charged forms of 2-APB show different characteristics during the insertion into membrane (Fig. 3c): for the neutral one, the favorable contributions from entropy is the main driving force along the permeation pathway; in contrast, for the charged one, the entropy component is unfavorable until the drug reach the hydrophobic core. Furthermore, the favorable entropy at the hydrophobic core of membrane is completely counterbalanced by a larger unfavorable internal energy, resulting a high free energy barrier (8 kcal/mol).

The variations of internal energy are compensated by the changes of entropy, as suggested by the apparent linear relationship between the two (Fig. 3d)^56,57^. It is worth noting that, regression of the data for the charged form shows a slope of −1.16, indicating an internal energy driven mechanism, while the neutral form has a slope of −0.78, suggesting that entropy may have a leading role in regulating the partition of 2-APB. Therefore, in addition to molecular polarity, entropy is another determinant that play a part in controlling the drug’s partition.

### Degrees of freedom of the charged 2-APB is restricted

Protonation of 2-APB has profound impacts on its internal degrees of freedom, and therefore accounting for entropy changes of the drug^58^.

**Figure 4:**
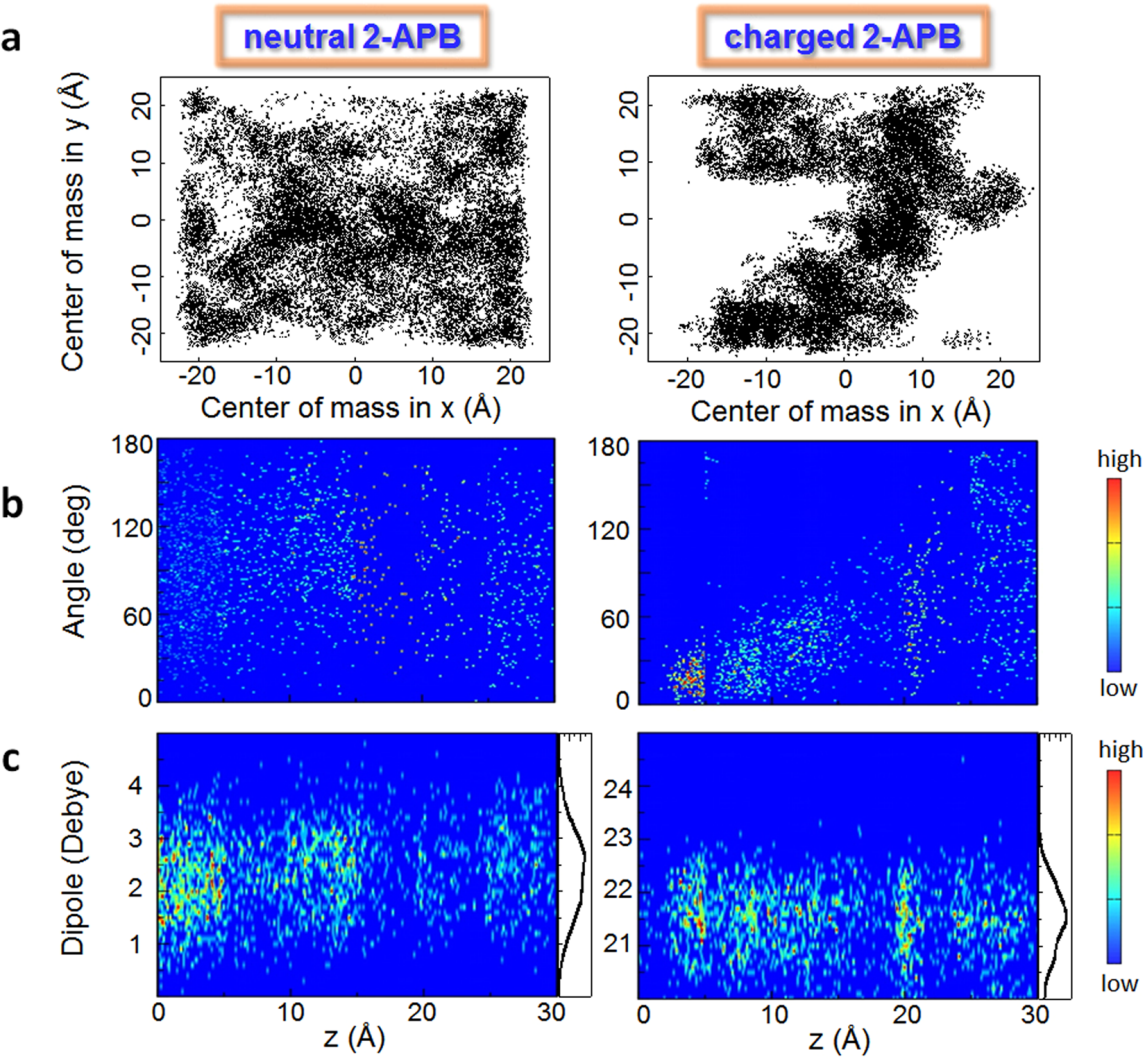
Difference in degrees of freedom leads to different entropy cost. (**a**) At the interior of membrane, the neutral 2-APB (left) diffuses freely to the whole region on the x-y plane, while the charged one (right) can only reach limited area, showing their different abilities in translational motion. At different position of membrane, the two forms also have distinct features with regard to the distribution of molecular orientation (**b**), where the neutral one (left) rotate more freely, while the orientation of charged one (right) is limited in the bilayer center, and the molecular dipole (**c**), that the neutral one (left) is boarder than the charged one (right) as indicated by the profile of the distribution shown on the right of each figure in (**c**).

Firstly, the charged form has lower mobility than that of the neutral one, mainly because its formation of specific/non-specific interactions with the environment during permeation, which causes a loss of configurational entropy. To be specific, at the interior of the membrane, the neutral form is capable of reaching a much wider area than that of the charged one (Fig. 4a), as can be seen from the trajectory projected on the plane perpendicular to the membrane normal. Similar case could be found for molecular rotation: the neutral form is likely to rotate freely in the system, as shown by the angle between the membrane normal and the dipole of the drug (Figs 2c and 4b), while the charged one has very limited orientations. In addition, the neutral form has higher conformational flexibility than that of the charged one, because broader distribution of dipole was observed (Fig. 4c). The reduced configurational degrees of freedom of ligand upon binding to protein and therefore the loss of entropy has been reported in literature^59–62^. Here we further demonstrate that the configurational of freedom is also critical for the membrane permeation of drug-like molecules, a step prior to protein binding.

In short, the neutral 2-APB is membrane permeable because it has favorable translational, rotational and conformational components of entropy. Presumably, the presence of SOC protein will decrease the mobility of bound 2-APB, due to the specific interactions. And more importantly, the difference of the conformational flexibility of 2-APB in different charged states is likely a key factor in regulating the function of SOC channel.

### Specific/non-specific interactions with the membrane distinguish the two forms of drug

Window based free energy calculations allow us to evaluate the interactions of the drug, where significant differences were observed for the different protonation states. Taking hydration of the amine group as an example, the charged form of drug has strong specific/non-specific interactions with either water molecule (Supplementary Figure S7a) or the ester group of the lipid (Fig. 2b) than that of the neutral one when they stay in the same depth of the membrane system. These interactions were found to be relevant to the flip-flop rate of small molecules inside the hydrophobic membrane core, a key step for their permeation through the membrane^63^.

### The protonation state affects the molecular polarity

**Figure 5:**
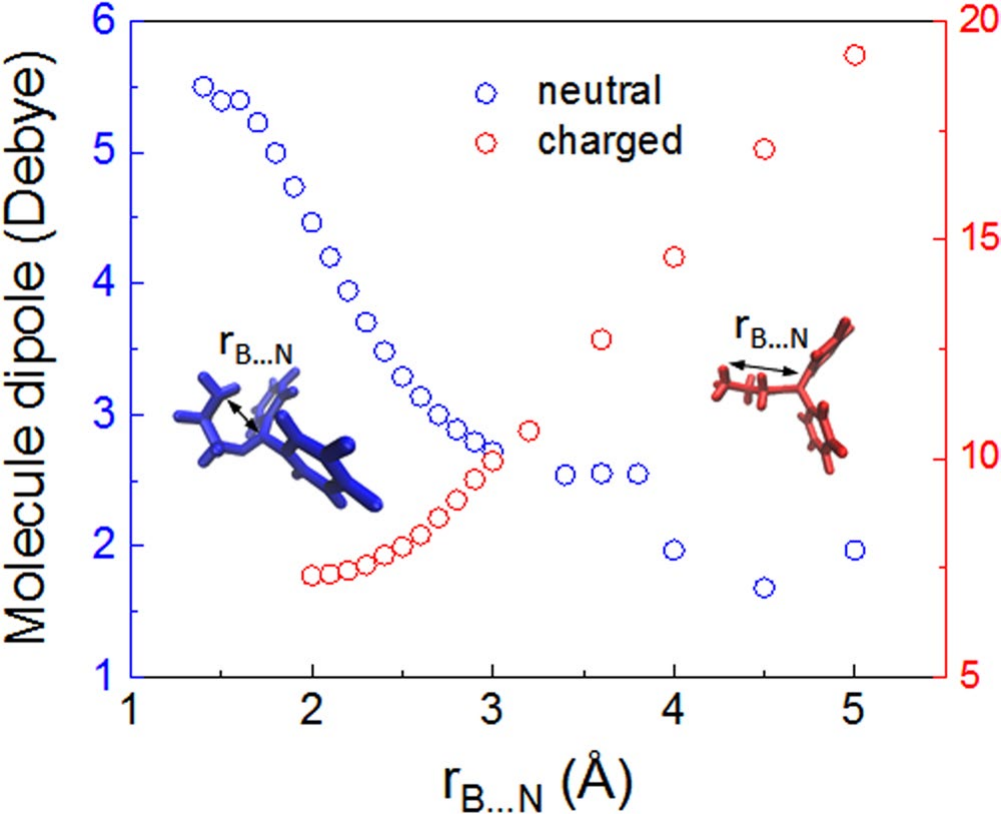
Correlation between molecular dipole and the B…N distance. Protonation on 2-APB affects it charge distribution, and therefore reserve the direction of molecular dipole.

The charge state greatly influences the polarity of 2-APB. As found in our previous work, the B…N interaction is critical for it function^16^. For neutral 2-APB, its dipole decreases when the B…N distance is increased, suggesting that the net dipole moment of the drug is partially counterbalanced by the dipole of B…N in the opposite direction (Fig. 5). To prove this conjecture, substituting two hydrogen atoms of the amino by two fluorine atoms leads to a somewhat smaller dipole moment of the molecule, while replacing the hydrogen atoms in *p*-benzene by two fluorine atoms leads to an increased dipole (Table S1). In contrast, when 2-APB is charged, the dipole moment increase as the increase of the distance between the B…N distance (Fig. 5), suggesting that protonation significantly affects 2-APB’s property by changing its charge distribution, and therefore its orientation and distributions in membrane (Fig. 2c). In the following section, we will show that entropy and polarity of drug synergistically affect its performance in membrane.

### Delicate Balance between the entropy and polarity for analogs of 2-APB

**Figure 6:**
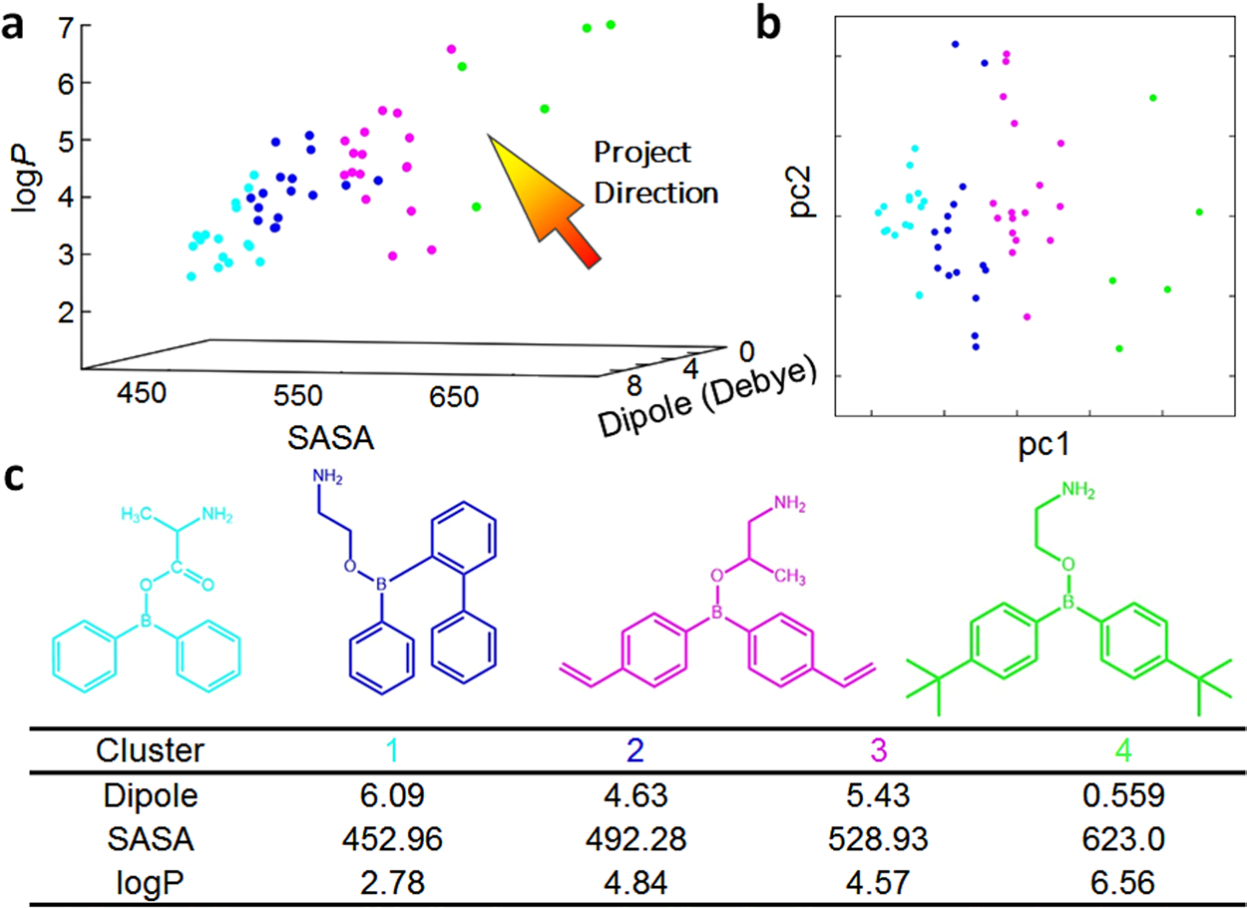
Delicate balance between entropy and polarity in 54 analogs of 2-APB. (**a**) 3-d plot of the solvent accessible surface area (SASA), dipole and log*P*. 4 groups are distinguished by different colors. The arrow shows the direction of project after dimension reduction. (**b**) 2-d plot obtained from 3-d by using PCA. (**c**) Representative structures in each group.

Based on the abovementioned information, comprehensive consideration for entropy and electrostatic factors are desired for the high-throughput screening of drug with higher efficacy. As a tentative attempt, we focus on 54 drug-like compounds based on the parent structure 2-APB’s backbone (Table S2). The 3-dimensional plot (Fig. 6a) of structures with different molecular size (entropy related), dipole (polarity related), and log*P*, clearly show the relationship among these variables: while all three factors are strongly coupled, delicate balance between volume and dipole was observed.

To have better understanding about different factors, density-based clustering method^64^ was used to divide these structures into 4 groups, which were mainly distinguished by molecular size. This is clearly demonstrated by the variable reduced 2- dimensional plot (Fig. 6b), which was achieved by using the principal component analysis (PCA): the first principal component mainly comes from molecular volume, and molecular dipole servers as the second principal component. In each group, the elements have similar size but quite different polarity (Fig. 6c), mainly because of the substitution with fluorine or tertiary butyl. Interestingly, fluorine was proposed to have potential ability to affect the docking drug-like compound in the binding pocket of protein^65^. Notably, good correlation between molecular volume and its log*P* was found, which well supports the previous conclusion that solvent accessible surface area was able to be used to empirically predict log*P* ^66^.

## Conclusion

In this work, we studied the permeation behavior of 2-aminoethoxydiphenyl borate (2-APB), a broad-spectrum modulator for a number of membrane proteins, by using MD simulations with polarization effect taken into account. Free energy calculations showed that the neutral form of 2-APB is permeable to the POPC lipid bilayer, while the permeation of the charged form is energetically unfavorable. The p*K*_a_ shift of 2-APB between the interior and the exterior of the membrane indicates the change of its protonation state during the permeation pathway. We further found that entropy has great impacts on 2-APB’s partition and transportation, mainly because of the different degrees of freedom of different protonated species. A survey on 54 derivatives with similar backbone to 2-APB showed that delicate balance between entropy and polarity plays an important role in drugs’ potency.

## Electronic supplementary material

Supplementary Information
