# Supplementary material for: Entropy and Polarity Control the Partition and Transportation of Drug-like Molecules in Biological Membrane
Source: Sci Rep. 2017 Dec 18;7:17749. doi: 10.1038/s41598-017-18012-7 (PMC5735159; doi:10.1038/s41598-017-18012-7)
Supplement: Supplementary file 1 — Supplementary Information [file 41598_2017_18012_MOESM1_ESM.pdf]

Electronic Supplementary Information

Entropy and Polarity Control the Partition and Transportation of Drug-like Molecules in Biological Membrane

Qiang Zhu[1,2], Yilin Lu[1], Xibing He[3], Tao Liu[1], Hongwei Chen[1], Fang Wang[1,4], Dong Zheng[2], Hao Dong[1,*], Jing Ma[2,*]

[1]*Kuang Yaming Honors School, Nanjing University, 210023, P. R. China;*

[2]*Institute of Theoretical and Computational Chemistry, School of Chemistry and Chemical Engineering, Nanjing University, 210023, P. R. China;*

[3]*School of Pharmacy, University of Pittsburgh, 3501 Terrace Street, Pittsburgh, PA 15213, USA;*

[4]*College of electronic information engineering, Sanjiang University, 210023, P. R. China.*

**Emails: donghao@nju.edu.cn; majing@nju.edu.cn*

Table of Contents

1. Flowchart of the procedure of the explicit polarizable model……………………….S2
2. Flowchart of the procedure of the implicit polarizable model……………………….S2
3. Figure of the assembled system……………………………………………………………..S3
4. Figure of charges derived from different protocols……………………………………..S3
5. Figure of the population using different protocols……………………………………..S4
6. Figure of the charges using different PCM models……………………………………..S4
7. Figure of the thermodynamic cycle for pK_a shift……………………………………..S5
8. Figure of the free energy under 3 different temperatures…………………………..S5
9. Figure of the radial distribution function…………………………………………………..S6
10. Computational methods…………………………………………………………………………S6
    1) parameterization……………………………………………………………………………..S7
    2) explicit polarizable model………………………………………………………………..S8
    3) implicit polarizable model………………………………………………………………..S8
    4) system setup……………………………………………………………………………………S8
    5) unbiased MD simulations………………………………………………………………..S8
    6) free energy calculation……………………………………………………………………..S9
    7) partition coefficient………………………………………………………………………..S9
    8) pK_a calculations………………………………………………………………………..S9
    9) properties of 54 drug-like molecule……………………………………………………S9
11. Table of dipole with substitution at different positions…………………………….S10
12. Table of properties of 54 drug-like molecules………………………………………..S11
13. Charge for the implicit model………………………………………………………………..S27
14. References……………………………………………………………………………………….S29

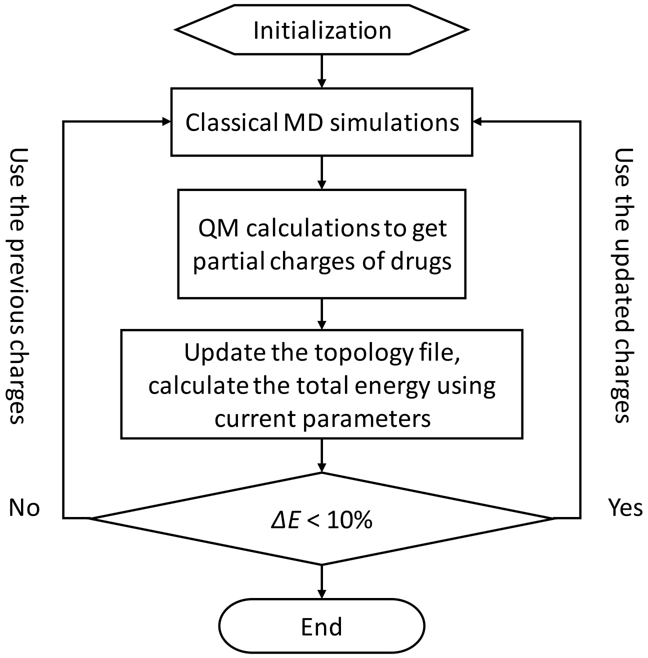

Scheme S1. Flowchart of the procedure of the explicit polarizable model.

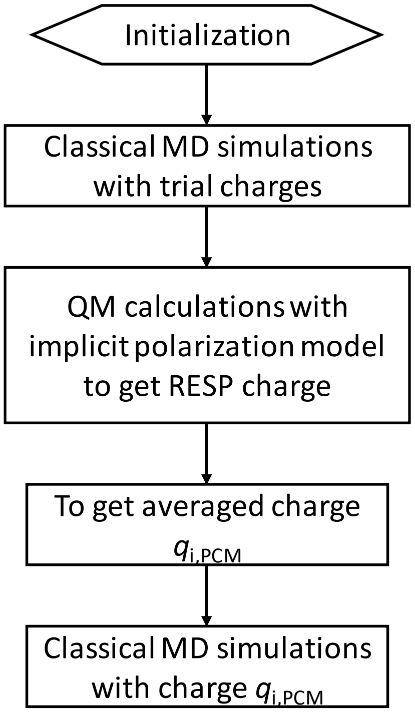

Scheme S2. Flowchart of the procedure of the implicit polarizable model.

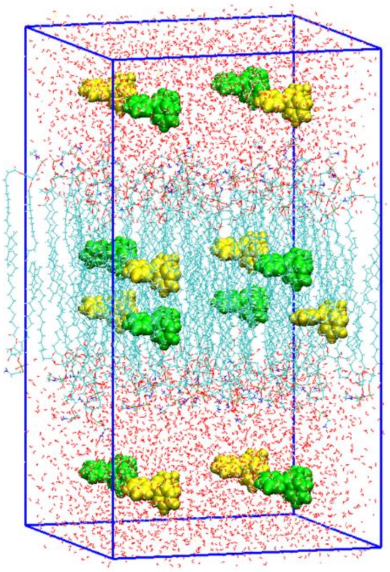

Figure S1. The initially assembled system. The yellow represents the neutral 2-APB, and the green denotes the charged one.

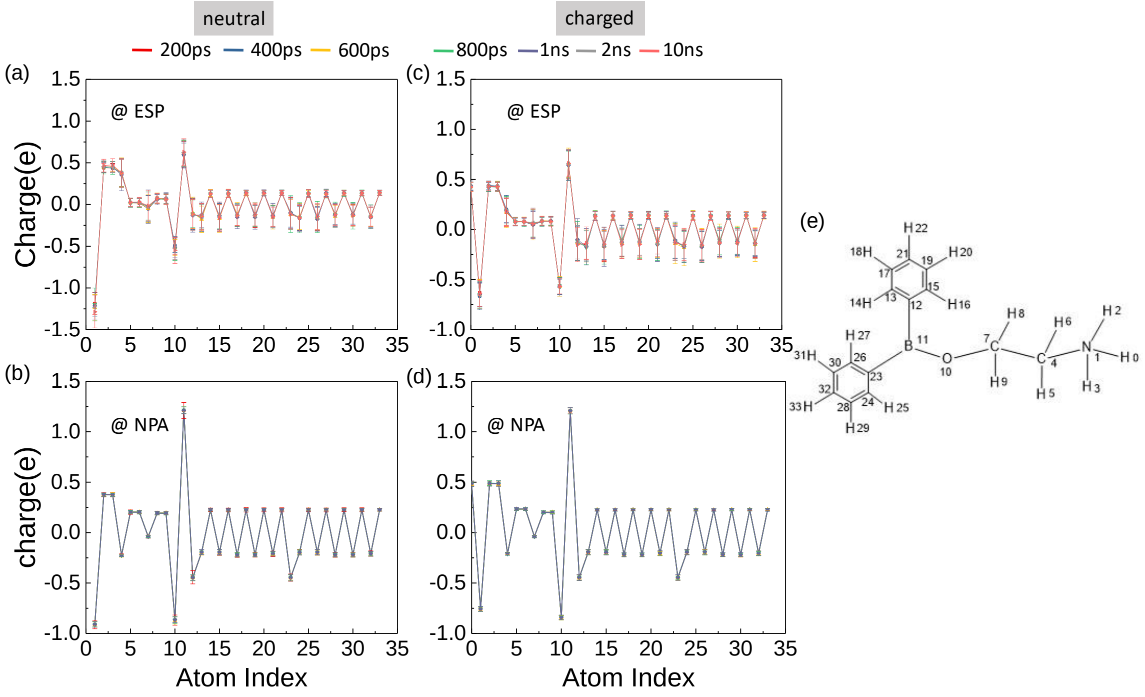

Figure S2. Partial change of both neural (left panel) and charged (right panel) 2-APB by using different protocols. (a) and (c) are EPS charges, (b) and (d) are NPA charges. X-axis represents the atom index shown in (e). Different lengths of interval got quite consistent partial charges, though the shorter one is computationally much more expensive.

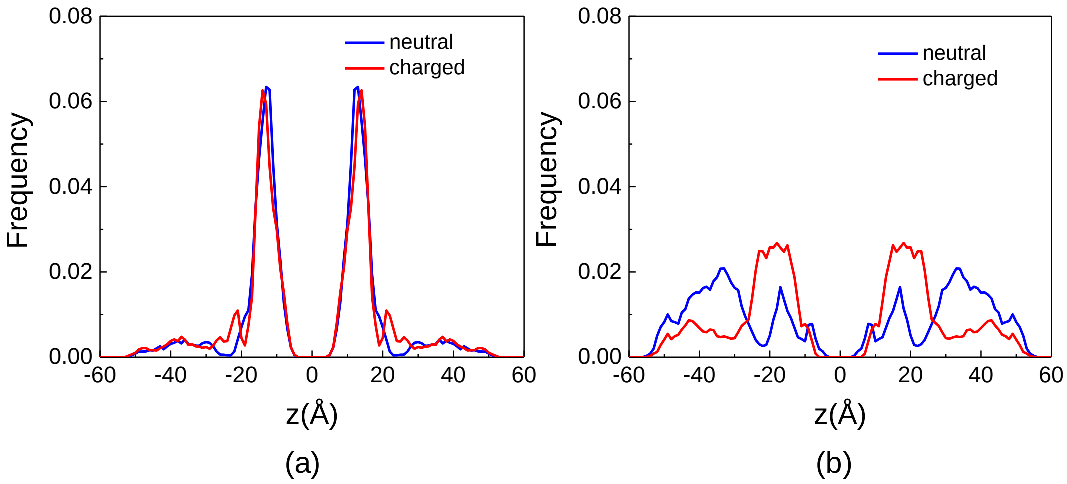

Figure S3. The population of neutral and charged forms of 2-APB as a function of distance from bilayer center using (a) ESP and (b) NPA. Systems are assumed to be symmetric, one half of the profile is calculated by averaging the two halves, the other half is the mirror of it. By comparing with the log*P* determined from experiments, the 2-APB was predicted to be less lipophilic by using NPA charge. Therefore, the performance of ESP charge is much better.

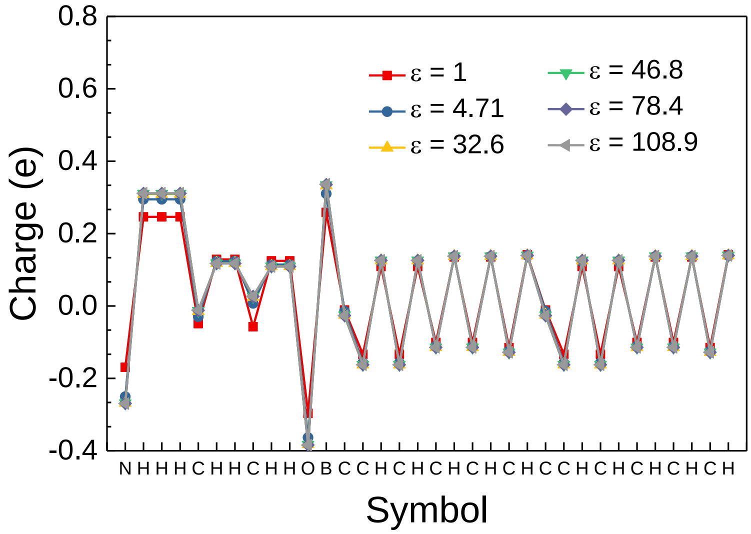

Figure S4. Partial charge of the charged 2-APB under different dielectric constant. The values 1, 4.71, 32.6, 46.8, 78.4, 108.9, of ε represent the air, chloroform, methanol, thionyl chloride, water and methanamide, respectively.

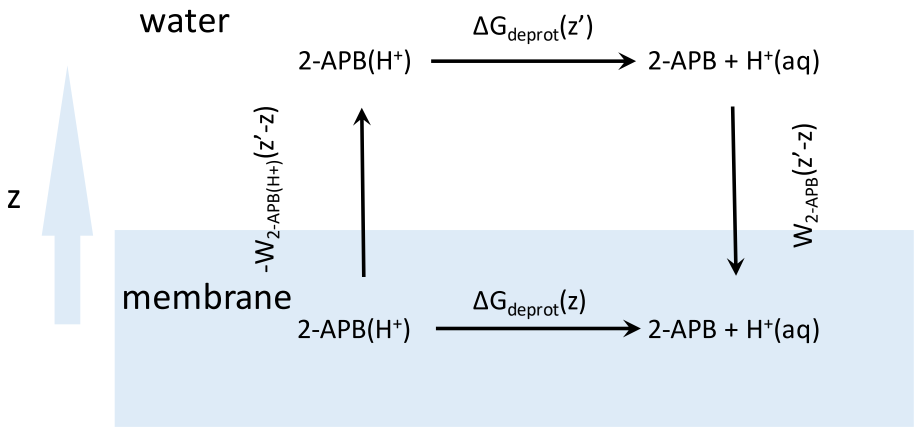

Figure S5. Thermodynamic cycle for the calculation of pK_a shift

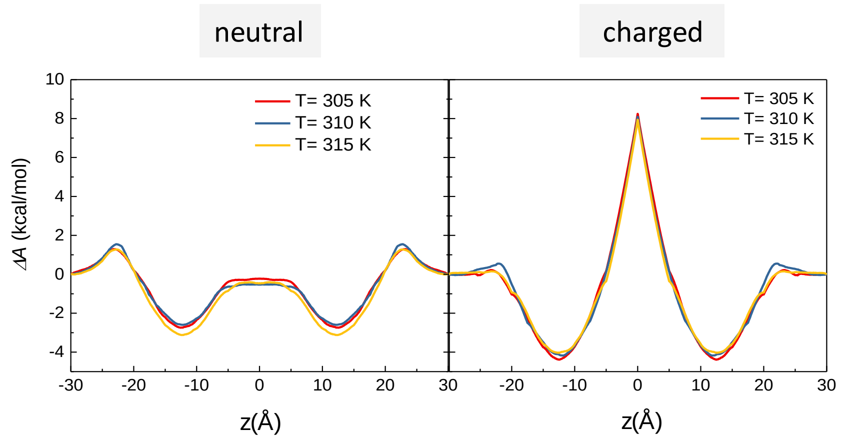

Figure S6. Temperature dependence of the free energies for the neutral (a) and charged (b) forms of 2-APB.

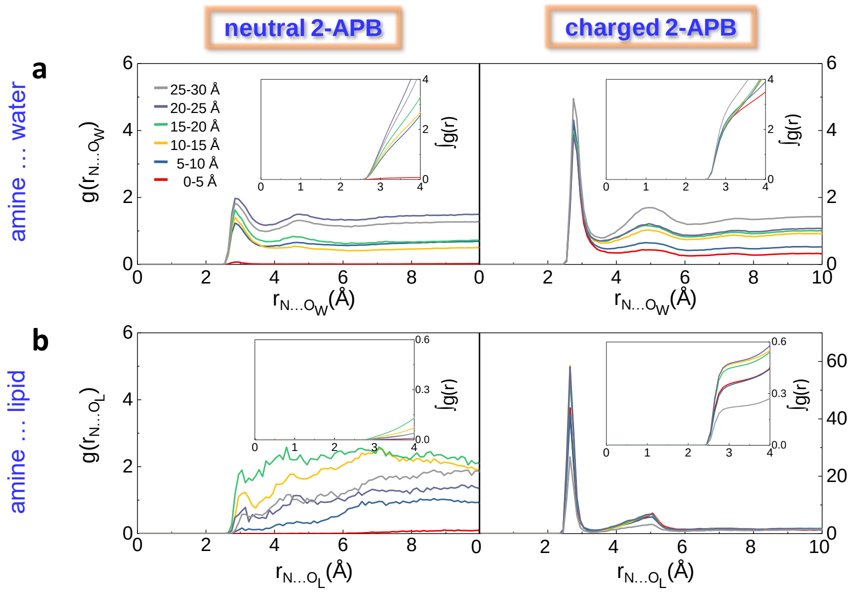

Figure S7. Radial distribution function between amine on 2-APB and oxygen on water (a) or ester (b).

Computational Methods:

(1) Parameterization for 2-APB with conventional CHARMM force field. To get the initial force field parameters for 2APB, CHARMM General Force Field (CGenFF) parameters were developed in a self-consistent fashion following previously described protocols[1-2] and initial parameters were generated from similar fragments. All empirical force field (MM) calculations were performed using the CHARMM program[3] and Quantum mechanical (QM) calculations were performed using the Gaussian09 program.[4] To prepare for QM target data, geometry optimizations and vibrational calculations were done at the MP2/6-31G(d) level.[5-6] The target QM frequencies were scaled by 0.943 to account for limitations in the level of theory.[7] QM level water interactions were used as target data for charge optimization, and water molecules in TIP3P geometry[8] were individually placed at optimized geometries interacting with hydrogen bond donors and acceptors in the model compound at its monomer MP2/6-31G(d) conformation. The interaction distance was optimized at the HF/6-31G(d) level with all other degrees of freedom fixed for each interacting pair. In order to yield parameters appropriate for the condensed phase simulation, the HF/6-31G* interaction energies were scaled by a factor of 1.16 and a difference of -0.2 Å in interaction distances between QM and MM was targeted for moderate to strong model compound-water interactions.[9-10] Relaxed potential energy scans (PES) were used to optimize dihedral parameters and were performed at the MP2/6-31G(d) level with the target dihedral angle(s) constrained at an increment step of 10°, while other degrees of freedom were fully optimized.

Gas phase MM energy minimizations were conducted using the conjugate gradient minimization method followed with the Newton-Raphson minimization algorithm to a gradient tolerance of 10^{-5} kcal/mol/Å using an infinite nonbonded cutoff distance. Vibrational calculations were done using the VIBRAN module and corresponding potential energy distribution (PED) analyses were carried out using MOLVIB module with internal valence coordinates as proposed by Pulay et al.[11] Such PED analyses were used to optimize force constants in bonded parameters. MM PES were performed by reading the QM geometries of all the scan points into CHARMM and harmonic restrains with force constant of 10,000

kcal/mol/radian were placed on the target dihedral angle(s) while all other degrees of freedom were minimized.

The parametrization started from optimizing atomic charges using QM water interactions and then move forward to reproduce minimized geometry and vibration analysis, and finally trying to reproduce QM PES profile in a self-consistent way. Due to lack of related experimental data, the van der Waals (VDW) parameters (epsilon and R_{min}) of the B atom were not specifically adjusted against the bulk properties of B-containing compounds. Fortunately in this special case, the B atom is buried at the center of bulky 2APB molecule. Even a wild range of change of the VDW parameters of B showed very limited affection on other properties like molecule-water interactions according to our test work.

(2) Explicit polarizable model. In this model, the general idea is that, the partial charges of a molecule were not fixed but were changeable in response to the local condition represented with background point charges.[12-14] As shown in Scheme 1, the procedure of the explicit-polarizable model is as follows:

(i) Run MD simulations with the conventional force field, and relax the system;

(ii) Based on the relaxed structure of the system, calculate the partial charge of a 2-APB molecule with QM methods, and the rest of the systems is represented as background point charge to mimic the polarization effect of the environment. For systems with multiple 2-APB molecules, each of them is treated with QM with all of the others being the environment. Considering the computational cost and accuracy, two cycles were performed to get the charge information of the each 2-APB molecules;

(iii) Update the partial charge of all 2-APB molecules, calculate the total energy with the new parameters, and compare it with the value obtained from the former parameters. If the energy difference is within 10%, the systems is believed to be stable. Otherwise, the former parameters will be used.

(iv) With a given interval, MD simulations are carried out with the parameters determined from the above step. Such cycle is repeated until the desired total time length of simulations is achieved.

The above procedure was implemented using a homemade Fortran code and TCL script embedded in the VMD.[15] *ab initio* quantum mechanical (QM) was used to get new partial charges. All the QM calculations were performed using the Gaussian 09 program package[4] at the M06-2X[16]/6-311++G(d,p) level of theory. Seemingly, two factors are critical in this explicit polarizable model. One is the partial charge, and the other is the interval used for updating charges. Extensive exams on these two factors were carried out.

Two kinds of population analysis, the natural population analysis (NPA) and the electro-static-potential (ESP) were tested. And seven independent MD simulations using different interval time to renew the atomic charge, i.e. 200ps, 400ps, 600ps, 800ps, 1000ps, 2ns, and 10ns, were carried out. The result was shown in Figures S2 and S3. Consequently, the combination of ESP charge with the interval of 10 ns was used.

(3) Implicit polarizable model. Different from the explicit polarizable model, in the present implicit one, the partial charge of the drug is fixed after parameterization. During the parameterization, the polarizable continuum model (PCM) was used to take the surrounding environment into consideration, and partial charge of the drug was derived from restrained fit of electrostatic potential[17] calculated with QM calculations (RESP charge), and the AMBER 16 package suite[18] was used. The protocol used to derive ESP partial charge is same as that we mentioned above. In addition, different values of ε were tested in this

work, as shown in Figure S4. We found that charges are nearly independent of the dielectric constant. Therefore, the ε value of water (78.4) was used to in this model. As shown in Scheme 2, the procedure contains the following steps:

(i) Run MD simulations with the conventional force field, and relax the system;

(ii) Based on the relaxed structure of the system, calculate the partial charge of a 2-APB molecule with QM methods for the subsequent RESP calculations, and the PCM was used to take the environment into account. Importantly, ensemble average of the trajectory was used to account for the redistribution of molecular dipole.

(iii) Use the partial charges obtained from step (ii) to run MD simulations.

(4) System setup. The lipid bilayer containing 91 POPC molecules was set up using the Package psfgen supplied by NAMD.[19] The membrane normal was oriented parallel to the z-axis of the simulation cell. 8 charged and 8 neutral forms of drugs were distributed evenly in the system (Figure S1), where half were in the membrane and half in the bulk water. To mimic the real physiological environment, 150 mM NaCl was added to the lipid bilayer. The total number of water, sodium, and chloride were 4281, 15, and 23 respectively, where the extra 8 anions were used to neutralize the system.

(5) Unbiased MD simulations. The molecular dynamics (MD) simulation was performed with the NAMD package suite.[19] CHARMM 36 force field[20] was used for lipids and ions and the TIP3P model for water.[8] The system was performed in the NPT ensemble at 310 K with 1 atm pressure. The electrostatic calculations were handled by the particle-mesh Ewald (PME)[21] with a 12 Å cutoff. Integration time step was set to be 2 fs with all bonds containing hydrogen held rigid using SHAKE algorithm[22]. Langevin dynamics was used to maintain temperature, while the pressure of the system was kept by a Nose-Hoover-Langevin piston,[23] except for the hydrogen atoms. During the simulations, the temperature was rescaled every 1000 integration steps, and the barostat oscillation time scale was set to be 100 fs.

For the system by using explicit polarizable model, 4 ns/day could be achieved by using NVIDIA GTX980Ti + Interl(R) Core(TM) i7-4790K(@4.0GHz). A toal of 200 ns trajectory was accumulated. For the implicit one, the performance is 12 ns/day by using the same machine, and we ran the MD simulations for 1 μs.

(6) Free energy calculations. Due to the relative long trajectory needed to get converged free energy profile, a system containing 59 POPC molecules, 2810 TIP3P water molecules was constructed for the biased MD simulation. In the system with neutral 2-APB, 7 sodium and 7 chlorides were added to mimic the physiological environment, while in the charged one, 7 sodium and 8 chlorides were added. The same force field parameters used for the unbiased MD simulations were applied for the calculation of potential of mean force. The simulation was performed with the NVT ensemble by using the adaptive biasing force method.[24-25] The distance between the center of mass of drug and all the phosphate atoms in the lipid (therefore the membrane center) was defined as a reaction coordinate. As the two leaflets are symmetric in membrane, the permeation free energy on one side was carried out. 6 non-overlapping windows ranging from 0 to 30 Å with an increase of 5 Å were set up. A bin width was set to 0.1 Å, and 200 steps for force samples was collected.

For the free energy decomposition, we run additional ABF calculations at 305 K and 315 K, respectively, with the same protocol mentioned above. To get converged free energy profiles, a total simulation time of 4.8 μs were collected for each temperature. For two protonation states at three different temperatures, a total of 28.8 μs trajectory was collected.

(7) Partition coefficient.

The definition of partition coefficient is shown in the following equation:

$$\log P_{oct/wat} = \log\left(\frac{[solute]_{oct}^{ionized} + [solute]_{oct}^{un-ionized}}{[solute]_{wat}^{ionized} + [solute]_{wat}^{un-ionized}}\right)$$

Where $[solute]_{oct}^{ionized}$ and $[solute]_{oct}^{un-ionized}$ represent the concentration of the drug resided in the octanol, $[solute]_{wat}^{ionized}$ and $[solute]_{wat}^{un-ionized}$ represent the one in the water.

(8) pK_a calculation.

$$pKa = pKa_{(ref)} + \frac{\Delta\Delta G_{deprot}}{2.3kT}\text{, and } \Delta\Delta G_{deprot} = W_{2-APB}(z'-z) - W_{2-APB(H^+)}(z'-z)$$

Where the $pKa_{(ref)}$ represents the value we obtained from the experiment in the bulk water.[26]

(9) Properties of 54 stuided drug-like molecules. Their partition coefficient (log*P*) and pK_a were all collected from the website of the sci-finder[27], the values of dipole moment were calculated using the G09[4] at the theory level of M06-2X/6-311++G(d,p), and values of Solvent Accessible Surface Area (SASA) were calculated by software VMD[15]. To classify the inherent relationship between these values, density-based clustering method[28] was used. Clustering by using K-means implemented in the scikit-learn[29] gave consistent results.

Table S1. Summary of the dipole moment with substituent group, with B…N distance constrained at 1.7 and 2.5 Å respectively.

	Structure	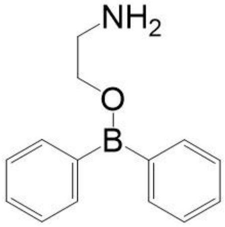	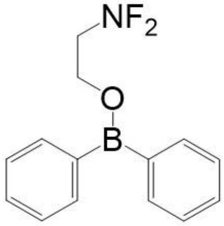	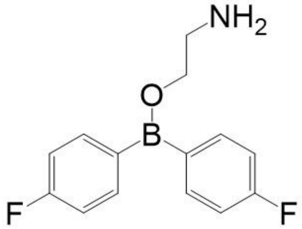
Dipole	$r_{B\ldots N}$ = 1.7 Å	5.23	5.17	6.91
(Debye)	$r_{B\ldots N}$ = 2.5 Å	3.29	2.67	4.52

Table S2. The summary of the properties of the studied 54 drug-like molecules, and their corresponding clustering labels.

CAS Registry Number	Structure	pK_a	Dipole (Debye)	SASA (angstrom^2)	log*P*	Clustering (log*P*-dipole-SASA)	Clustering (log*P*-pKa-SASA)
1028-93-9	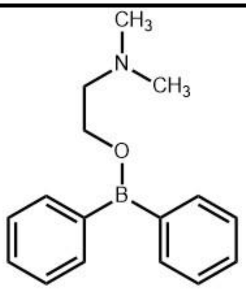	8.44±0.28	1.45	455.5	3.602±0.464	1	3
2262-43-3	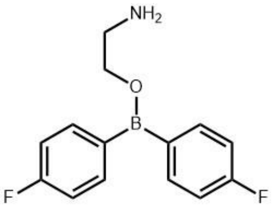	8±0.1	2.63	445.6	3.182±1.070	1	3
13488-41-0	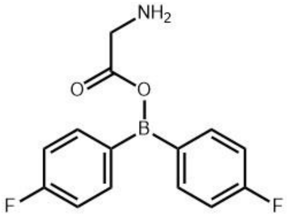	6.58±0.29	6.07	456.2	2.679±1.076	1	3

84008-17-3	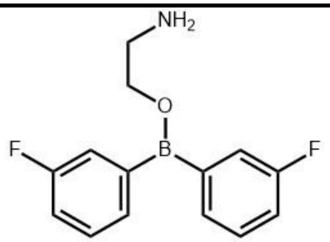	7.9±1.07	7.72	462.5	3.182±1.070	1	3
125689-54-5	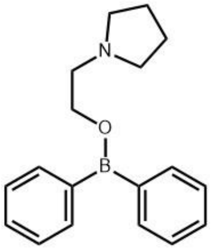	9.4±0.2	5.37	465.6	4.177±0.487	1	3
1475-86-1	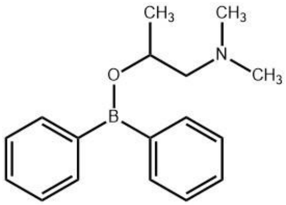	8.6±0.28	5.53	463.6	3.956±0.468	1	3
14335-29-6	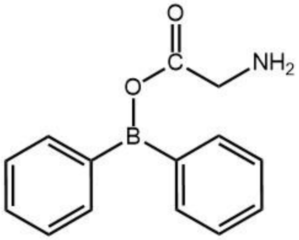	6.61±0.29	6.05	434.2	2.425±0.454	1	3

22858-33-9	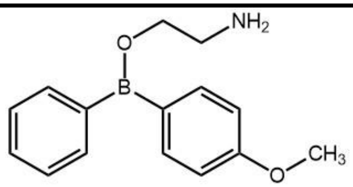	8.04±0.1	4.56	456.0	2.921±0.722	1	3
26584-69-0	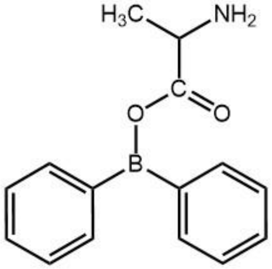	7.07±0.29	6.09	453.0	2.779±0.459	1	3
60288-72-4	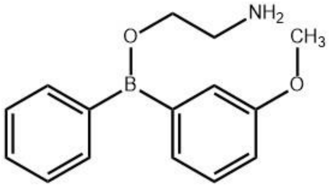	8.04±0.1	5.18	461.6	2.921±0.722	1	3
67457-14-1	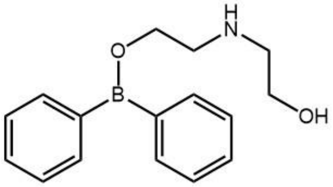	14.74±0.1	4.08	459.5	2.593±0.493	1	3
84008-13-9	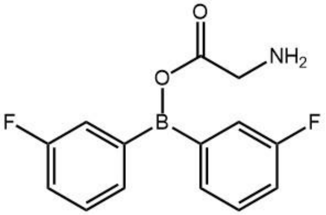	6.52±0.29	7.72	462.5	2.679±1.076	1	3

97881-33-9	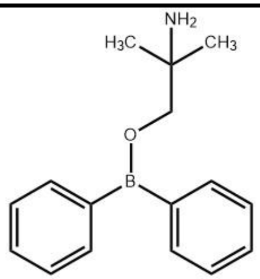	9.31±0.1	5.46	455.6	3.692±0.448	1	3
124383-91-1	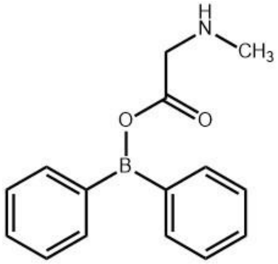	6.86±0.1	6.48	438.5	2.98±0.49	1	3
1028414-48-3	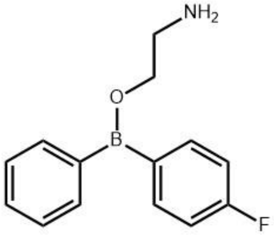	8.01±0.1	5.86	438.0	8.01±0.1	1	3
87439-18-7	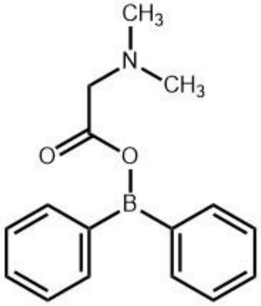	6.58±0.28	6.42	440.3	3.161±0.486	1	3

1181750-35-5	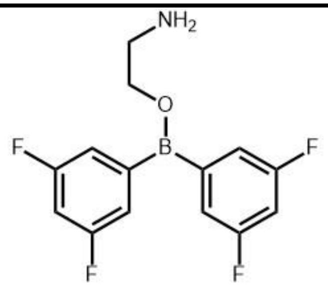	7.75±0.1	6.79	488.1	3.334±1.435	3	0
97881-34-0	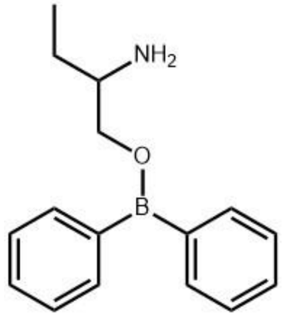	8.13±0.1	1.1074	487.1	3.791±0.435	3	0
2024-45-5	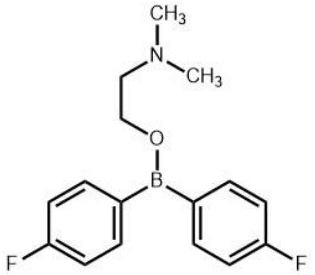	8.42±0.28	6.89	475.1	3.856±1.079	3	0

2284-67-5	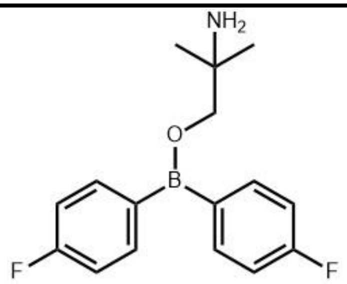	9.30±0.10	6.96	482.8	3.946±1.075	3	0
1181750-29-7	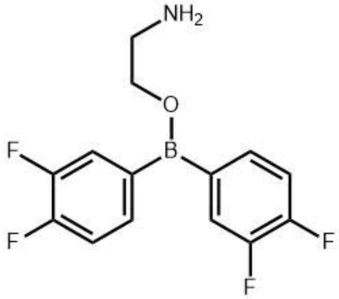	7.70±0.10	6.35	475.2	3.436±1.437	3	0
17303-77-4	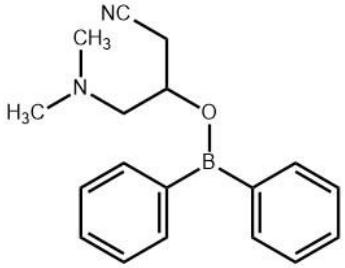	8.12±0.28	8.46	501.4	3.433±0.498	3	0
82491-90-5	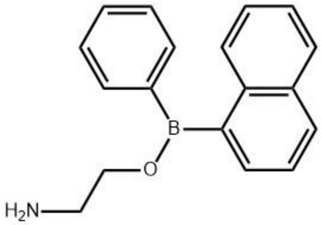	8.03±0.1	5.14	486.1	4.112±0.431	3	0

98438-45-0	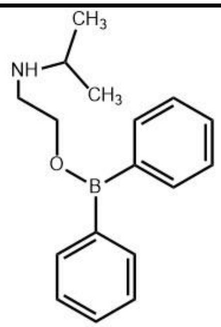	8.77±0.1	5.5	481.9	4.155±0.459	3	0
13488-25-0	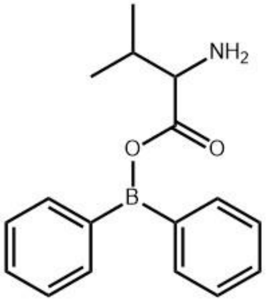	7.15±0.33	6.01	473.0	3.642±0.464	3	0
100261-27-6	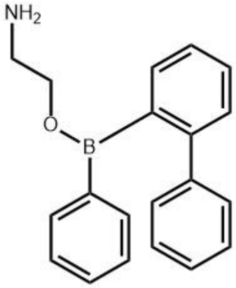	8.00±0.1	4.63	492.3	4.842±0.735	3	0

1181751-85-8	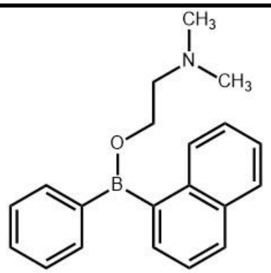	8.44±0.28	5.87	481.9	4.786±0.465	3	0
52936-90-0	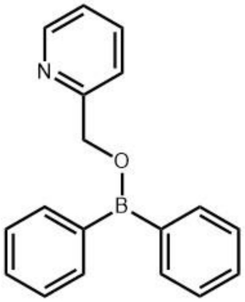	3.69±0.12	1.29	507.2	3.893±0.427	3	0
515158-41-5	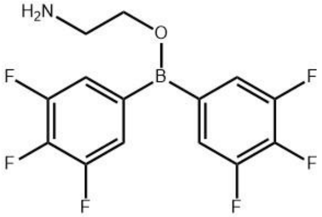	7.55±0.1	8.2	500.7	3.589±1.538	3	0
1181750-36-6	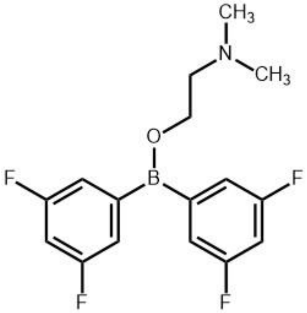	8.30±0.28	7.26	501.3	4.009±1.437	3	0

13488-43-2	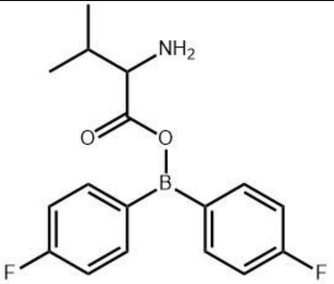	7.13±0.33	6.46	508.0	3.896±1.079	3	0
37763-56-7	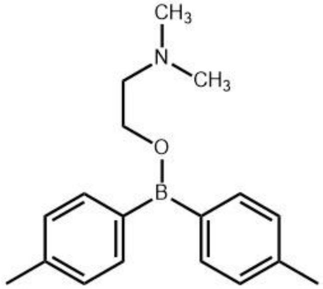	8.47±0.28	6.39	506.3	4.688±1.011	3	0
1379680-33-7	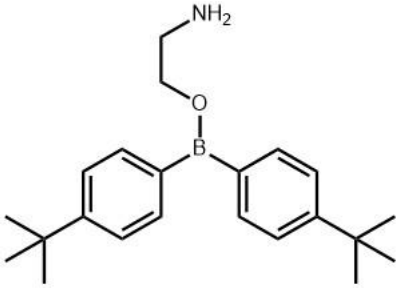	8.09±0.1	0.56	623.1	6.562±1.011	2	1

1181751-06-3	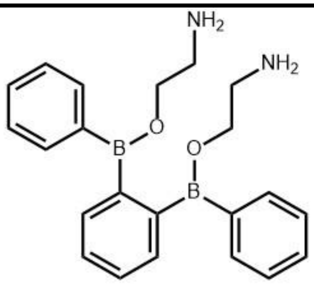	8.25±0.10	5.44	595.4	3.679±0.724	2	1
1816300-97-6	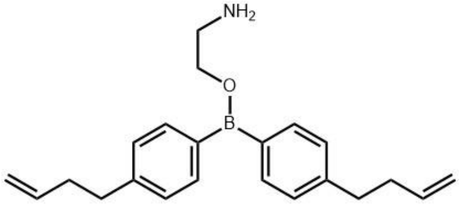	8.09±0.10	5.17	633.0	5.392±1.017	2	1
102032-41-7	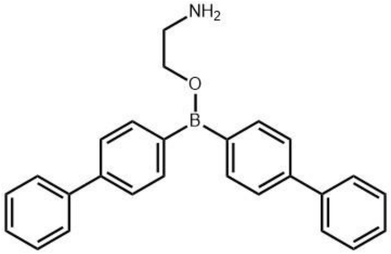	7.98±0.10	2.96	654.9	6.757±1.031	2	1
87439-20-1	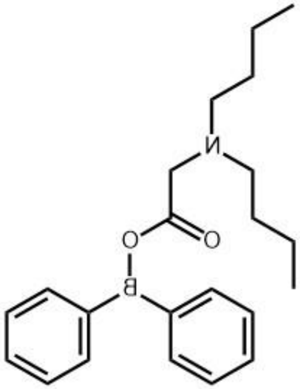	6.74±0.5	7.16	599.9	6.218±0.487	2	1

110155-67-4	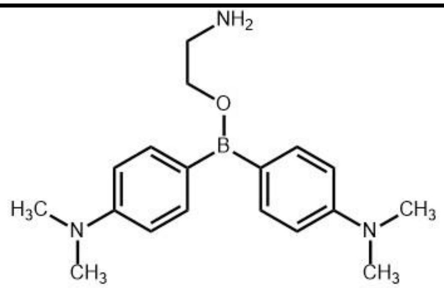	8.03±0.1	4.08	559.3	2.845±1.018	0	2
515157-54-7	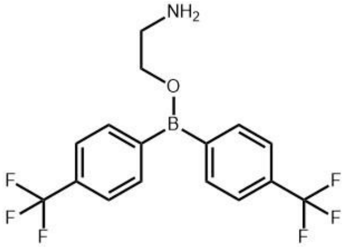	7.84±0.10	5.08	552.5	4.348±1.044	0	2
117052-53-6	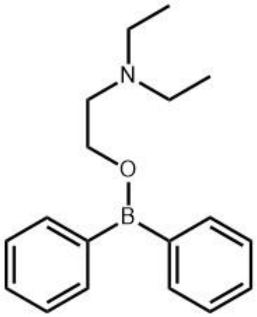	9.4±0.25	0.87	522.3	4.621±0.464	0	2

97440-37-4	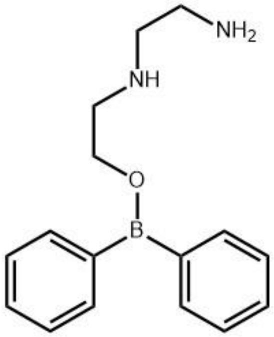	9.6±0.1	1.90	520.2	2.609±0.558	0	2
515157-62-7	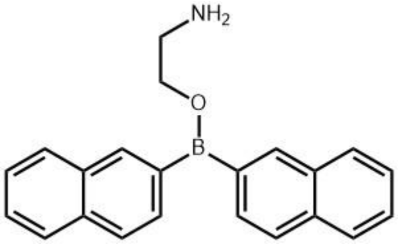	8.03±0.10	4.67	535.1	5.296±0.433	0	2
111385-59-2	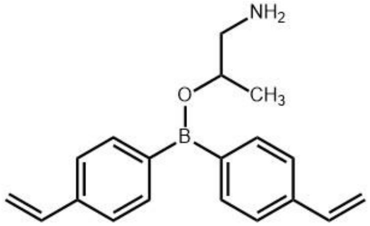	8.07±0.1	5.43	528.93	4.569±1.009	0	2
25386-69-0	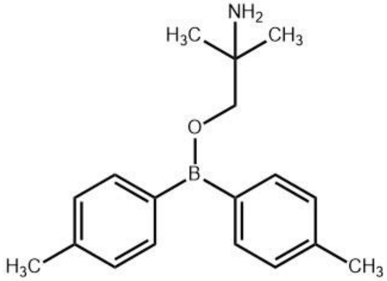	9.34±0.1	5.07	516.2	4.778±1.006	0	2

1593938-93-2	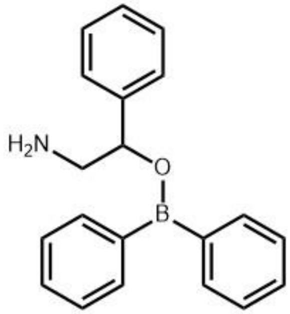	7.29±0.1	4.72	513.2	4.163±0.443	0	2
17303-78-5	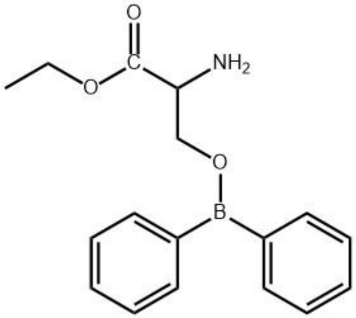	5.42±0.33	3.78	545.2	3.5±0.507	0	2
1181751-87-0	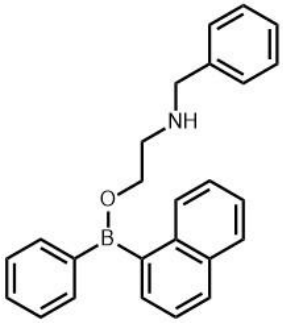	8.06±0.19	2.61	559.7	6.271±0.480	0	2

87439-21-2	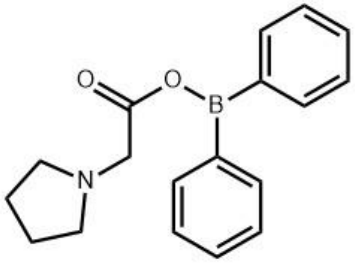	7.77±0.2	4.75	525.9	3.746±0.506	0	2
100261-29-8	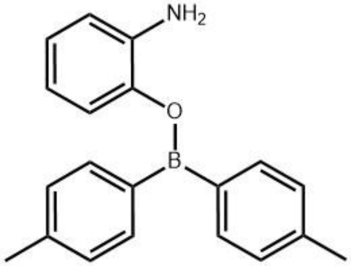	3.37±0.1	2.54	527.8	5.141±0.999	0	2
287193-27-5	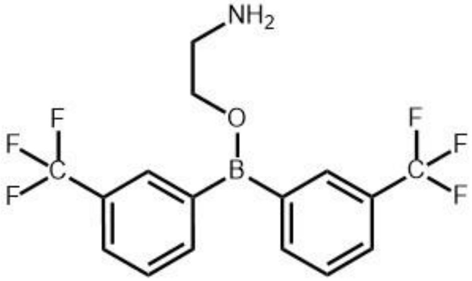	7.94±0.1	7.23	536.5	4.348±1.044	0	2
1593939-00-4	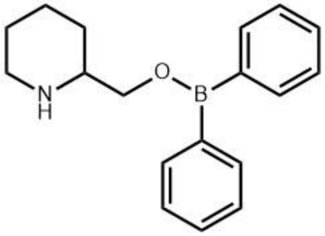	9.42±0.1	1.04	521.9	4.113±0.485	0	2

2884-08-4	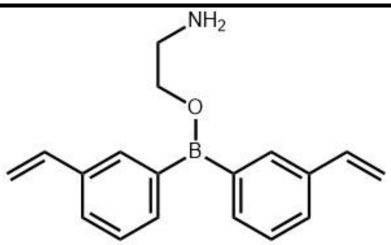	8.12±0.10	5.27	526.5	4.216±1.007	0	2
1593938-97-6	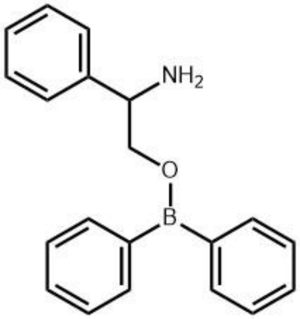	8.02±0.10	5.78	526.4	4.605±0.455	0	2
99269-70-2	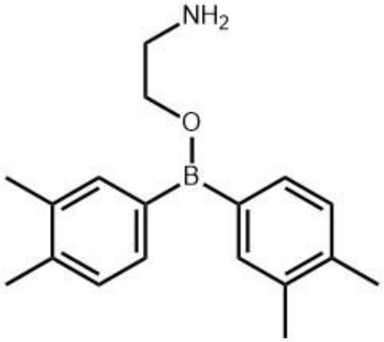	8.15±0.10	4.93	526.5	4.930±1.403	0	2

The charge for the charged 2-APB of the implicit model:

					Atom type	charge	mass	
1	M1	1	AMPB	N	NG3P3	-0.323084	14.0070	0
2	M1	1	AMPB	H01	HGP2	0.34081	1.0080	0
3	M1	1	AMPB	H02	HGP2	0.34081	1.0080	0
4	M1	1	AMPB	H03	HGP2	0.34081	1.0080	0
5	M1	1	AMPB	C1	CG324	0.056728	12.0110	0
6	M1	1	AMPB	H11	HGA2	0.105597	1.0080	0
7	M1	1	AMPB	H12	HGA2	0.105597	1.0080	0
8	M1	1	AMPB	C2	CG321	0.02139	12.0110	0
9	M1	1	AMPB	H21	HGA2	0.095805	1.0080	0
10	M1	1	AMPB	H22	HGA2	0.095805	1.0080	0
11	M1	1	AMPB	O	OG301	-0.450048	15.9994	0
12	M1	1	AMPB	B	BG331	0.567271	10.8110	0
13	M1	1	AMPB	C3	CG2R61	-0.145245	12.0110	0
14	M1	1	AMPB	C41	CG2R61	-0.139305	12.0110	0
15	M1	1	AMPB	H41	HGR61	0.119882	1.0080	0
16	M1	1	AMPB	C42	CG2R61	-0.139305	12.0110	0
17	M1	1	AMPB	H42	HGR61	0.119882	1.0080	0
18	M1	1	AMPB	C51	CG2R61	-0.11006	12.0110	0
19	M1	1	AMPB	H51	HGR61	0.128849	1.0080	0
20	M1	1	AMPB	C52	CG2R61	-0.11006	12.0110	0
21	M1	1	AMPB	H52	HGR61	0.128849	1.0080	0
22	M1	1	AMPB	C6	CG2R61	-0.134226	12.0110	0
23	M1	1	AMPB	H6	HGR61	0.131994	1.0080	0
24	M1	1	AMPB	C7	CG2R61	-0.145245	12.0110	0
25	M1	1	AMPB	C81	CG2R61	-0.139305	12.0110	0
26	M1	1	AMPB	H81	HGR61	0.119882	1.0080	0
27	M1	1	AMPB	C82	CG2R61	-0.139305	12.0110	0
28	M1	1	AMPB	H82	HGR61	0.119882	1.0080	0
29	M1	1	AMPB	C91	CG2R61	-0.11006	12.0110	0
30	M1	1	AMPB	H91	HGR61	0.128849	1.0080	0
31	M1	1	AMPB	C92	CG2R61	-0.11006	12.0110	0
32	M1	1	AMPB	H92	HGR61	0.128849	1.0080	0
33	M1	1	AMPB	C10	CG2R61	-0.134226	12.0110	0
34	M1	1	AMPB	H10	HGR61	0.131994	1.0080	0

The charge for the neutral 2-APB of implicit model:

273	N1	1	2APB	N	NG321	-1.027554	14.0070	0
274	N1	1	2APB	H01	HGPAM2	0.392481	1.0080	0
275	N1	1	2APB	H02	HGPAM2	0.392481	1.0080	0
276	N1	1	2APB	C1	CG321	0.239730	12.0110	0
277	N1	1	2APB	H11	HGA2	0.037773	1.0080	0
278	N1	1	2APB	H12	HGA2	0.037773	1.0080	0
279	N1	1	2APB	C2	CG321	-0.021405	12.0110	0
280	N1	1	2APB	H21	HGA2	0.073322	1.0080	0

281	N1	1	2APB	H22	HGA2	0.073322	1.0080	0
282	N1	1	2APB	O	OG301	-0.422705	15.9994	0
283	N1	1	2APB	B	BG331	0.519427	10.8110	0
284	N1	1	2APB	C3	CG2R61	-0.119064	12.0110	0
285	N1	1	2APB	C41	CG2R61	-0.140724	12.0110	0
286	N1	1	2APB	H41	HGR61	0.116715	1.0080	0
287	N1	1	2APB	C42	CG2R61	-0.140724	12.0110	0
288	N1	1	2APB	H42	HGR61	0.116715	1.0080	0
289	N1	1	2APB	C51	CG2R61	-0.115531	12.0110	0
290	N1	1	2APB	H51	HGR61	0.126157	1.0080	0
291	N1	1	2APB	C52	CG2R61	-0.115531	12.0110	0
292	N1	1	2APB	H52	HGR61	0.126157	1.0080	0
293	N1	1	2APB	C6	CG2R61	-0.130973	12.0110	0
294	N1	1	2APB	H6	HGR61	0.129481	1.0080	0
295	N1	1	2APB	C7	CG2R61	-0.119064	12.0110	0
296	N1	1	2APB	C81	CG2R61	-0.140724	12.0110	0
297	N1	1	2APB	H81	HGR61	0.116715	1.0080	0
298	N1	1	2APB	C82	CG2R61	-0.140724	12.0110	0
299	N1	1	2APB	H82	HGR61	0.116715	1.0080	0
300	N1	1	2APB	C91	CG2R61	-0.115531	12.0110	0
301	N1	1	2APB	H91	HGR61	0.126157	1.0080	0
302	N1	1	2APB	C92	CG2R61	-0.115531	12.0110	0
303	N1	1	2APB	H92	HGR61	0.126157	1.0080	0
304	N1	1	2APB	C10	CG2R61	-0.130973	12.0110	0
305	N1	1	2APB	H10	HGR61	0.129481	1.0080	0

References

(1) Vanommeslaeghe, K.; Hatcher, E.; Acharya, C.; Kundu, S.; Zhong, S.; Shim, J.; Darian, E.; Guvench, O.; Lopes, P.; Vorobyov, I.; Mackerell, A. D., Jr., *J. Comput. Chem.* **2010,** *31*, 671.
(2) Yu, W.; He, X.; Vanommeslaeghe, K.; MacKerell, A. D., Jr., *J. Comput. Chem.* **2012,** *33*, 2451.
(3) Brooks, B. R.; Brooks, C. L., 3rd; Mackerell, A. D., Jr.; Nilsson, L.; Petrella, R. J.; Roux, B.; Won, Y.; Archontis, G.; Bartels, C.; Boresch, S.; Caflisch, A.; Caves, L.; Cui, Q.; Dinner, A. R.; Feig, M.; Fischer, S.; Gao, J.; Hodoscek, M.; Im, W.; Kuczera, K.; Lazaridis, T.; Ma, J.; Ovchinnikov, V.; Paci, E.; Pastor, R. W.; Post, C. B.; Pu, J. Z.; Schaefer, M.; Tidor, B.; Venable, R. M.; Woodcock, H. L.; Wu, X.; Yang, W.; York, D. M.; Karplus, M., *J. Comput. Chem.* **2009,** *30*, 1545.
(4) Frisch, M. J.; Trucks, G. W.; Schgel, H. B.; Scuseria, G. E.; Robb, M. A.; Cheeseman, J. R.; Scalmani, G.; Barone, V., *Gaussian 09, revision D. 01; Gaussian, Inc.: Wallingford, CT* **2009**.
(5) Moller, C.; Plesset, M. S., *Phys. Rev.* **1934,** *46*, 0618.
(6) Hariharan, P. C.; Pople, J. A., *Theor. Chem. Acc.* **1973,** *28*, 213.
(7) Scott, A. P.; Radom, L., *J. Phys. Chem.* **1996,** *100*, 16502.
(8) Jorgensen, W. L.; Chandrasekhar, J.; Madura, J. D.; Impey, R. W.; Klein, M. L., *J. Chem. Phys.* **1983,** *79*, 926.
(9) Mackerell, A. D., *J. Comput. Chem.* **2004,** *25*, 1584.
(10) Mackerell, A. D.; Karplus, M., *J. Phys. Chem.* **1991,** *95*, 10559.
(11) Pulay, P.; Fogarasi, G.; Pang, F.; Boggs, J. E., *J. Am. Chem. Soc.* **1979,** *101*, 2550.
(12) Yan, X.; Jiang, N.; Ma, J., *Sci. China, Ser. B* **2009,** *52*, 1925.
(13) Jiang, N.; Ma, J., *Journal of Chemical Physics* **2012,** *136*.
(14) Wang, X.; Yan, T.; Ma, J., *Int. J. Quantum Chem.* **2015,** *115*, 545.
(15) Humphrey, W.; Dalke, A.; Schulten, K., *J. Molec. Graphics Modell.* **1996,** *14*, 33.
(16) Zhao, Y.; Truhlar, D. G., *Theor. Chem. Acc.* **2008,** *120*, 215.
(17) Cieplak, P.; Caldwell, J.; Kollman, P., *J. Comput. Chem.* **2001,** *22*, 1048.
(18) Case, D. A.; Cerutti, D. S.; T. E. Cheatham, I.; Darden, T. A.; Duke, R. E.; Giese, T. J.; Gohlke, H.; Goetz, A. W.; Greene, D.; Homeyer, N.; Izadi, S.; Kovalenko, A.; Lee, T. S.; LeGrand, S.; Li, P.; Lin, C.; Liu, J.; Luchko, T.; Luo, R.; Mermelstein, D.; Merz, K. M.; Monard, G.; Nguyen, H.; Omelyan, I.; Onufriev, A.; Pan, F.; Qi, R.; Roe, D. R.; Roitberg, A.; Sagui, C.; Simmerling, C. L.; Botello-Smith, W. M.; Swails, J.; Walker, R. C.; Wang, J.; Wolf, R. M.; Wu, X.; Xiao, L.; York, D. M.; Kollman, P. A., *University of California, San Francisco* **AMBER 2017**.
(19) Philips, J. C.; Braun, R.; Wang, W.; Gumbart, J.; Tajkhorshid, E.; Villa, E.; Chipot, C.; Skeel, R. D.; Kale, L.; Schulten, K., *J. Comput. Chem.* **2005,** *26*, 1781.
(20) Klauda, J. B.; Venable, R. M.; Freites, J. A.; O'Connor, J. W.; Mackerell, A. D.; Pastor, R. W., *J. Phys. Chem. B* **2010,** *114*, 7830.
(21) Darden, T.; York, D.; Pedersen, L., *J. Chem. Phys.* **1993,** *98*, 10089.
(22) Allen, M. P.; Tildesley, D. J., *Oxford University Press, NY* **1989**, 95.
(23) Feller, S. E.; Zhang, Y.; Pastor, R. W.; Brooks, B. R., *J. Chem. Phys.* **1995,** *103*, 4613.
(24) Darve, E.; Rodriguez-Gomez, D.; Pohorille, A., *J. Chem. Phys.* **2008,** *128*, 144120.
(25) Henin, J.; Fiorin, G.; Chipot, C.; Klein, M. L., *J. Chem. Theory Comput.* **2010,** *6*, 35.
(26) Tao, L.; Harris, A. L., *Mol. Pharmacol.* **2007,** *71*, 570.
(27) *SciFinder, version 2017; Chemical Abstracts Service: Columbus, OH, 2017; LogP value; calculated using ACD/Labs software, version 11.02; ACD/Labs 1994-2017*.
(28) Rodriguez, A.; Laio, A., *Science* **2014,** *344*, 1492.

(29) Pedregosa, F.; Varoquaux, G.; Gramfort, a.; Michel, V.; Thirion, B.; Grisel, O.; Blondel, M.; Prettenhofer, P.; Weiss, R.; Fubourg, V.; Vanderplas, J.; Passos, A.; Brucher, M.; Perrot, M.; Duchesnay, E., *J MACH LEARN RES* **2011,** *12*, 2825.
